# Expression of p53 protein in high-grade gastroenteropancreatic neuroendocrine carcinoma

**DOI:** 10.1371/journal.pone.0187667

**Published:** 2017-11-07

**Authors:** Abir Salwa Ali, Malin Grönberg, Birgitte Federspiel, Jean-Yves Scoazec, Geir Olav Hjortland, Henning Grønbæk, Morten Ladekarl, Seppo W. Langer, Staffan Welin, Lene Weber Vestermark, Johanna Arola, Pia Österlund, Ulrich Knigge, Halfdan Sorbye, Lars Grimelius, Eva Tiensuu Janson

**Affiliations:** 1 Department of Medical Sciences, Section of Endocrine Oncology, Uppsala University, Uppsala, Sweden; 2 Department of Pathology, Copenhagen University Hospital Rigshospitalet, Copenhagen, Denmark; 3 Department of Biopathology, Institut Gustave Roussy, Villejuif, France; 4 Department of Oncology, Oslo University, Oslo, Norway; 5 Department of Hepatology & Gastroenterology, Aarhus university Hospital, Aarhus, Denmark; 6 Department of Oncology, Aarhus University Hospital, Aarhus, Denmark; 7 Department of Oncology, Copenhagen University Hospital Rigshospitalet, Copenhagen, Denmark; 8 Department of Oncology, Odense University Hospital, Odense, Denmark; 9 Pathology, HUSLAB, University of Helsinki and Helsinki University Hospital, Helsinki, Finland; 10 Department of Oncology, Helsinki University Hospital and Helsinki University, Helsinki Finland; 11 Department of Oncology, Tampere University Hospital, Tampere, Finland; 12 Department of Surgery C and Endocrinology PE, Rigshospitalet, Faculty of Health Science, University of Copenhagen, Copenhagen, Denmark; 13 Department of Oncology, Haukeland University Hospital and Department of Clinical Science, University of Bergen, Bergen, Norway; 14 Department of Immunology, Genetics and Pathology, Uppsala University, Uppsala, Sweden; Heinrich-Heine-Universitat Dusseldorf, GERMANY

## Abstract

**Background:**

Gastroenteropancreatic neuroendocrine carcinomas (GEP-NECs) are aggressive, rapidly proliferating tumors. Therapeutic response to current chemotherapy regimens is usually short lasting. The aim of this study was to examine the expression and potential clinical importance of immunoreactive p53 protein in GEP-NEC.

**Materials and methods:**

Tumor tissues from 124 GEP-NEC patients with locally advanced or metastatic disease treated with platinum-based chemotherapy were collected from Nordic centers and clinical data were obtained from the Nordic NEC register. Tumor proliferation rate and differentiation were re-evaluated. All specimens were immunostained for p53 protein using a commercially available monoclonal antibody. Kaplan-Meier curves and cox regression analyses were used to assess progression-free survival (PFS) and overall survival (OS).

**Results:**

All tumor tissues were immunoreactive for either one or both neuroendocrine biomarkers (chromogranin A and synaptophysin) and Ki67 index was >20% in all cases. p53 immunoreactivity was only shown in 39% of the cases and was not found to be a prognostic marker for the whole cohort. However, p53 immunoreactivity was correlated with shorter PFS in patients with colorectal tumors (HR = 2.1, p = 0.03) in a univariate analysis as well as to poorer PFS (HR = 2.6, p = 0.03) and OS (HR = 3.4, p = 0.02) in patients with colorectal tumors with distant metastases, a correlation which remained significant in the multivariate analyses.

**Conclusion:**

In this cohort of GEP-NEC patients, p53 expression could not be correlated with clinical outcome. However, in patients with colorectal NECs, p53 expression was correlated with shorter PFS and OS. Further studies are needed to establish the role of immunoreactive p53 as a prognostic marker for GEP-NEC patients.

## Introduction

Gastroenteropancreatic neuroendocrine carcinomas (GEP-NECs) are defined by WHO as poorly differentiated neuroendocrine neoplasms (NENs). Their proliferative capacity is high, with Ki67 proliferation index >20% and/or mitoses >20 per 2 mm^2^ [1].

GEP-NECs account for approximately 35–55% of all extra-pulmonary NECs. They are mainly located in the esophagus, stomach, pancreas, colon and rectum, but in 30% of cases, they present as tumors of unknown primary location [2].

The present WHO 2010 classification for NENs G3 has been debated for not being optimal as it assumes that all G3 tumors are poorly differentiated. Furthermore, the WHO G3 group includes all tumors with Ki67 index above 20% as one disease entity. In recent publications, the presence of tumors that are well-differentiated, but with Ki67 index >20% and with a better prognosis than poorly differentiated GEP-NECs was demonstrated [3, 4]. However, assessing the degree of differentiation may be challenging and there is a need of biomarkers which may help to discriminate between GEP-NEC patients with better and worse prognosis.

There is a reported increase in incidence of GEP-NECs over the years, but there is still a lack of effective treatment resulting in persistent poor survival for these patients [5, 6]. In the Nordic NEC study of 305 patients, median overall survival (OS) was 11 months for patients treated with chemotherapy and 1 month for untreated patients. Pancreatic tumors showed a median OS of 15 months, while rectal and colon tumors had median OS of 10 and 8 months, respectively; indicating that OS differs with primary tumor locations [2, 7]. Other reported factors indicating a better prognosis are Ki67 index <55%, normal serum lactate dehydrogenase (LDH) and platelet count as well as good performance status [2].

Platinum-based chemotherapy has been used as a first-line treatment for GEP-NECs since the nineties and the Nordic, European and North American Societies of neuroendocrine tumors (NETs) recommend combination chemotherapy with cisplatin/carboplatin and etoposide [8–10]. In the recent Nordic NEC study, GEP-NEC patients were shown to respond differently to chemotherapy when divided in different groups by Ki67 index and there is a need for new and better biomarkers to predict therapeutic response and survival. GEP-NECs with Ki67 index <55% showed a lower objective response rate (yet the same disease control rate, (DCR)) to chemotherapy compared to those with a higher Ki67 index, but still had a longer survival [2].

*TP53* is a known tumor suppressor gene normally present in all human cells and the p53 pathway is usually activated by different types of stress signals due to e.g. DNA damage [11]. The tumor suppressing characteristics of wild type (WT) p53 is essential for genome stability and cell cycle arrest, and in the presence of DNA damage, WT p53 may induce cell repair and/or give rise to apoptosis [6]. Mutations in *TP53* are common and occur in many cancer types, including NEC: 70–100% of tumor cells have been shown to be mutated in high grade pulmonary NECs [12]. Further, *TP53* mutations are associated with poorer clinical outcome, treatment resistance and higher degree of metastases in different types of cancer [13–15]. Mutations in *TP53* have been shown to result in an immunohistochemically detectable expression of the p53 protein; since the mutated protein is not degraded, it accumulates into tumor cell nuclei [16].

A few studies have investigated the immunohistochemical expression of p53 protein in carcinomas and most of them have shown heterogeneity in the outcome [6, 17, 18]. The aim of this study was to examine the immunohistochemical expression of p53 protein in a large cohort of GEP-NEC tumors collected retrospectively, including patients managed according to the same therapeutic principles.

We hypothesized that immunohistochemical expression of p53 protein is associated with shorter progression-free survival (PFS) and OS and might be of prognostic relevance in GEP-NEC patients.

## Materials and methods

### Patient and tumor characteristics

This cohort included patients diagnosed with poorly differentiated GEP-NEC with a primary tumor located in the gastrointestinal tract or a cancer of unknown primary (CUP). CUP was defined as NEC with predominant abdominal metastases but where no primary tumor could be identified.

Tumor specimens were collected retrospectively based on availability from the Nordic NEC Study, resulting in 124 GEP-NEC patients treated with a platinum-based chemotherapy at the Nordic Centers, and diagnosed 1999–2011. Clinical data was obtained from the Nordic NEC register [2].

Formalin-fixed paraffin-embedded (FFPE) material included: 40 needle biopsies, 20 surgical biopsies and 64 surgical specimens. All tumors were immunoreactive (IR) for CgA and/or synaptophysin and all tumors had Ki67 index >20%. An endocrine pathologist (LG) recalculated the frequency of Ki67 IR tumor cells in all tumor samples and the morphological differentiation (well vs. poorly) was re-assessed by a panel of experienced neuroendocrine pathologists.

Fifty-seven tumor specimens were retrieved from primary tumors and 67 from metastases. Exclusion criteria were Ki67 index <20% and the diagnose of a mixed adenocarcinoma-neuroendocrine carcinomas (MANEC) based on the WHO definition describing these tumors as having an exocrine and endocrine component where the neuroendocrine component is present in at least 30% of the tumor [19].

Patients were divided into groups depending on location of the primary tumor: esophagus, stomach, pancreas, colon and rectum, or CUP. Four tumors were located in the esophagus, 11 in the stomach, 28 in the pancreas, 31 in colon and 17 in rectum. In 33 cases, the primary tumor was unknown.

### Clinical variables used in statistical analysis

The clinical variables chosen to be investigated in the statistical analyses included age (median age 60 years), Ki67 index, LDH, therapeutic response (evaluated according to the RECIST criteria) and performance status (according to the Eastern Cooperative Oncology Group consensus (ECOG)).

Specimen size was included to evaluate if larger specimens yielded more tumor cells IR for p53. A sub-analysis for PFS and OS was done for patients with distant metastases for each group of primary tumor location.

### Immunohistochemistry

FFPE tissue specimens were cut into approximately 4-µm thick sections and attached to positively charged glass slides (Superfrost Plus, Menzel Gläser, Braunschweig, Germany).

Before immunostaining, the sections were treated in a pressure cooker reaching maximum temperature of 121°C using Tris-HCL buffered saline, pH 9.0 as retrieval solution. The sections were incubated with a primary monoclonal antibody (anti-p53, clone DO-7, Dako, Glostrup, Denmark) at room temperature for 30 minutes (dilution 1:100). A polymer-detection system was used (EnVision Plus-HRP, Dako, Glostrup, Denmark) according to manufacturer’s instructions. Diaminobenzidine was used as chromogen.

### Quantification

The Ki67 index was calculated in a light-microscope at a magnification of x40, with a square grid in one of the ocular to facilitate the cell counting. At least 2000 tumor cells were counted in the areas with the highest tumor cell proliferation. In small biopsies, containing less than 2000 tumor cells, all tumor cells were counted. The Ki67 index was expressed as the percentage of IR tumor cells.

The presence of p53 immunoreactivity was semi-quantitatively estimated (in percent) by assessing the area of IR tumor cells vs the total tumor area by light-microscopy at a magnification of x40. Ten percent or more p53 nuclear IR tumor cells in the tumor area was considered as positive outcome based on the results from a previous study performed with the same antibody [20].

Photographs were taken using a Zeiss Observer Z1 microscope and the Axiovision software (Carl Zeiss, Gottingen, Germany).

### Controls

Colon adenocarcinoma tissue, with a known *TP53* mutation, was used as a positive control, and omission of the primary antibody was used as a negative control.

### Statistical analyses

The defined event was death from any cause. PFS was defined as the time between date of first treatment and date of tumor progression and OS was defined as time from diagnosis of locally advanced or metastatic disease until date of death; or if event was not found, censored at date of last observation.

Kaplan-Meier plots were used for PFS and OS analysis, and the log-rank test was used to compare curves separated according to expression of p53. Cox proportional regression was performed for the estimation of hazard ratios (HRs) and confidence intervals (CIs).

Spearman correlation was used to assess the correlation of p53 protein expression to clinicopathological variables. For the statistical analyses all variables were dichotomized: p53 IR vs. non-IR, age ≤60 years vs. >60 years, Ki67 ≥55% vs. ≤55% LDH normal vs. high and performance status ECOG 0+1 vs. 2+3.

All statistical analyses were performed using IBM SPSS statistics software (v22, USA).

### Ethics

Local ethics committees in the Nordic countries from which tissue samples were collected approved the research protocol.

The study was approved and the need for consent was waived by the local ethics committee, Regionala etikprövningsnämnden (EPN, Dnr2008/397), in Uppsala, Sweden.

## Results

### Immunoreactivity and staining patterns in tumor samples

**Fig 1:**
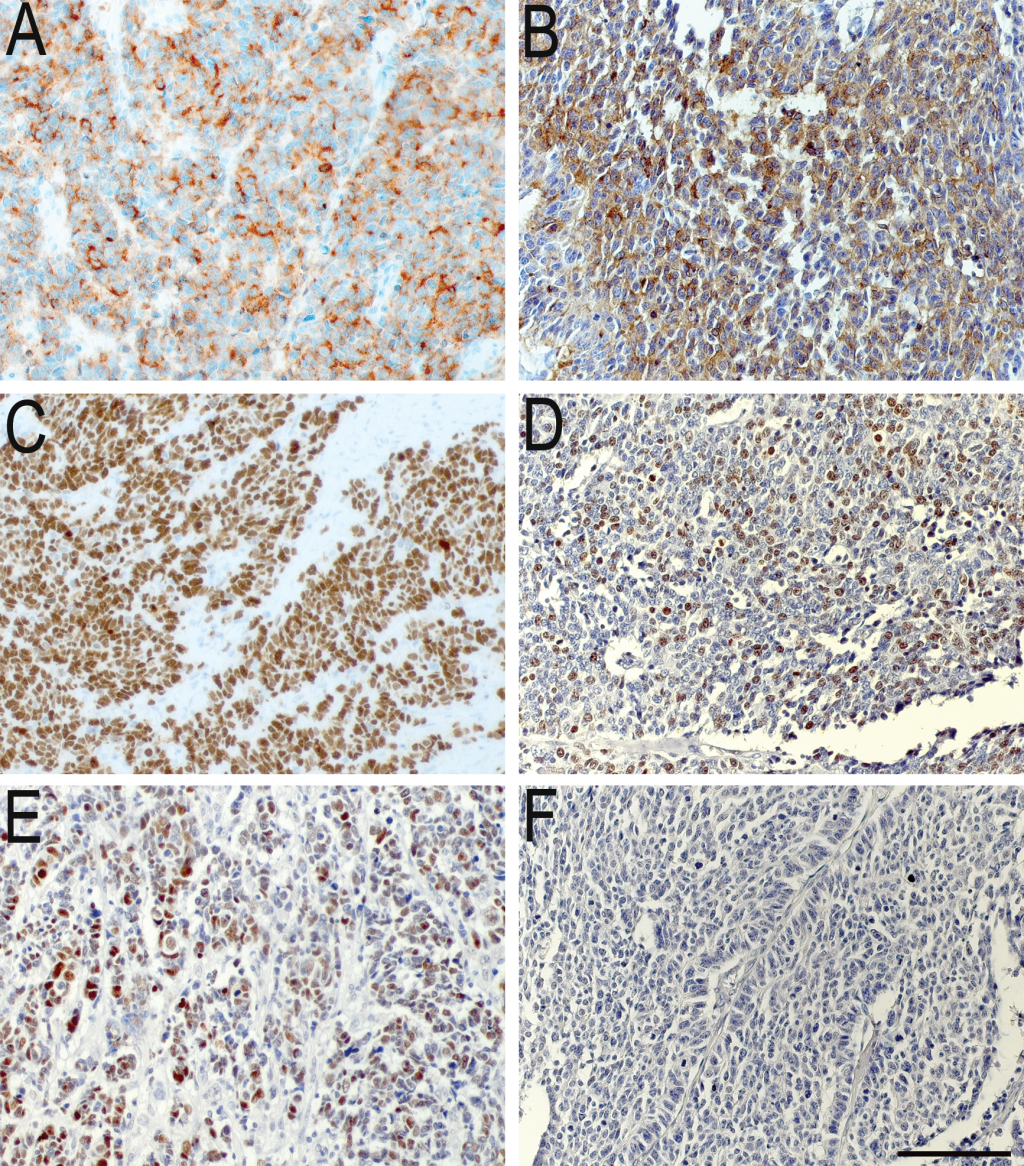
Representative images of immunostainings. (A, B) Immunoreactivity for chromogranin A, (C, D) Ki67 and (E, F) p53. The left panel demonstrates staining of a pancreatic primary tumor. The right panel shows the respective staining from a rectal primary tumor. Scale bar = 100 μm.

Of the 124 tumors stained, 39% (n = 48) were p53 IR (Table 1). The frequency of p53 IR cells in the tumors varied between 20–100%. The apparent intensity of nuclear staining was strong in the majority of tumor cells. All tumors were verified to be poorly differentiated, by an experienced endocrine pathologist. Representative images from p53 immunostainings are shown in Fig 1.

**Table 1:** Tumour characteristics.

	Total, *n*	p53 IR, *n*	p53 non-IR, *n*
**All tumors *(%)***	124	48 (39%)	76 (61%)
**Primary**			
Esophagus	4	3	1
Stomach	11	4	7
Pancreas	28	11	17
CUP	33	9	24
Colon	31	14	17
Rectum	17	7	10
**Disease stage**			
Local	3	2	1
Regional	24	8	16
Distant	97	38	59
**Ki67**			
<55%	46	12	34
>55%	78	36	42

CUP, cancer with unknown primary; IR, immunoreactive.

Colon primary was the tumor group with most frequently p53 IR cells followed by pancreas, CUP, rectum, stomach and esophagus. In both the p53 IR and p53 non-IR groups, approximately 80% had distant metastases and Ki67 was >55% in a majority of patients in both groups (Table 1).

**Fig 2:**
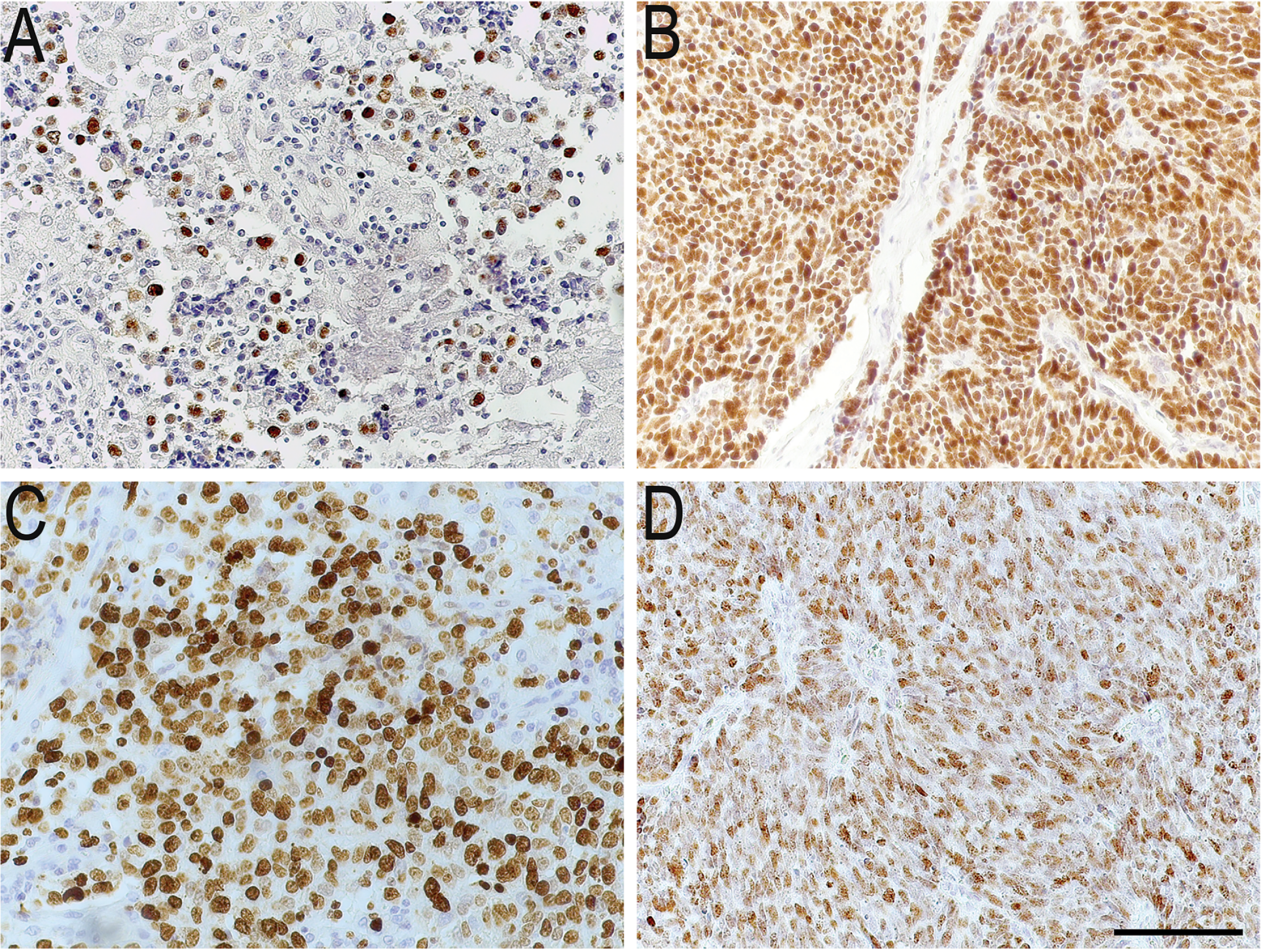
Immunohistochemical images of scattered and dense staining pattern. Scattered and dense staining pattern for two p53 immunoreactive tumors. (A) Scattered type. Single immunoreactive cells spread out in the whole tumor sample. (B) Dense type. Widespread immunoreactivity of the entire tumor specimen. (C) and (D) represent Ki67 for each tumor respectively. Scale bar = 100 μm.

Based on the p53 staining, two different patterns could be distinguished: one with scattered cells and the other with densely packed cells. Five of the 48 p53 IR tumors (10%) showed a scattered pattern, where 20–40% of the tumor cells were p53 IR. The remaining 43 (90%) had a homogenous pattern where 60–100% of the tumor cells were IR (Fig 2A–2D).

### Correlations of p53 immunoreactivity with clinicopathological variables

Statistical analyses of the whole cohort with all clinical variables dichotomized showed that p53 immunoreactivity was positively correlated with Ki67 with a higher frequency of p53 IR cells for patients with Ki67 index above 55%. The tumor specimen size correlated positively with p53 immunoreactivity in the whole cohort i.e. large specimens were more often IR. There was no correlation between p53 expression and small cell or large cell morphology.

For patients with colorectal tumors, a positive correlation was found between p53 immunoreactivity and performance status showing that those with p53 IR tumors had a poorer performance status compared to those with non-IR tumors. For patients with colorectal tumors and distant metastases, p53 immunoreactivity correlated negatively with treatment response. No significant result was obtained for correlation with response from sub-analysis of patients with metastatic primaries in esophagus, the gastric mucosa or pancreatic or in the CUP subgroup.

Spearman’s correlations are presented in Table 2.

**Table 2:** p53 protein expression in relation to clinicopathological variables.

	N	ρ	p-value
**Whole cohort**			
Age	121	-0.11	0.25
Ki67	124	0.20	0.03*
LDH	112	0.01	0.91
Performance status^●^	122	0.04	0.69
Response	117	0.09	0.36
Specimen size	124	0.23	0.01**
**Esophagus, stomach and pancreatic primaries**			
Age	42	-0.07	0.66
Ki67	43	0.16	0.29
LDH	39	0.03	0.87
Performance status^●^	41	-0.12	0.46
Response	41	0.21	0.18
Specimen size	43	0.40	0.01**
**CUP**			
Age	32	-0.09	0.62
Ki67	33	0.22	0.21
LDH	28	-0.18	0.35
Performance status^●^	33	-0.20	0.25
Response	32	0.17	0.33
Specimen size	33	0.05	0.77
**Colorectal primaries**			
Age	47	-0.20	0.21
Ki67	48	0.15	0.27
LDH	45	0.19	0.21
Performance status^●^	48	0.28	0.05*
Response	44	0.23	0.13
Specimen size	48	0.21	0.15
**Colorectal primaries with distant metastases**			
Age	35	-0.12	0.60
Ki67	36	0.10	0.53
LDH	34	0.31	0.07
Performance status^●^	36	0.37	0.03*
Response	33	0.40	0.02*
Specimen size	36	0.05	0.77

ρ, Spearman’s correlation test coefficient; CUP, cancer with unknown primary; LDH, lactate dehydrogenase.

^●^ Eastern Cooperative Oncology Group consensus (ECOG) for performance status.

*correlation is significant at the 0.05 level

**correlation is significant at the 0.01 level

Dichotomized variables: p53 IR vs. non-IR, age ≤60 yrs vs. >60 yrs, Ki67 ≥55% vs. ≤55% LDH normal vs. high and performance status ECOG 0+1 vs. 2+3.

### Association between p53 protein expression and prognosis

**Fig 3:**
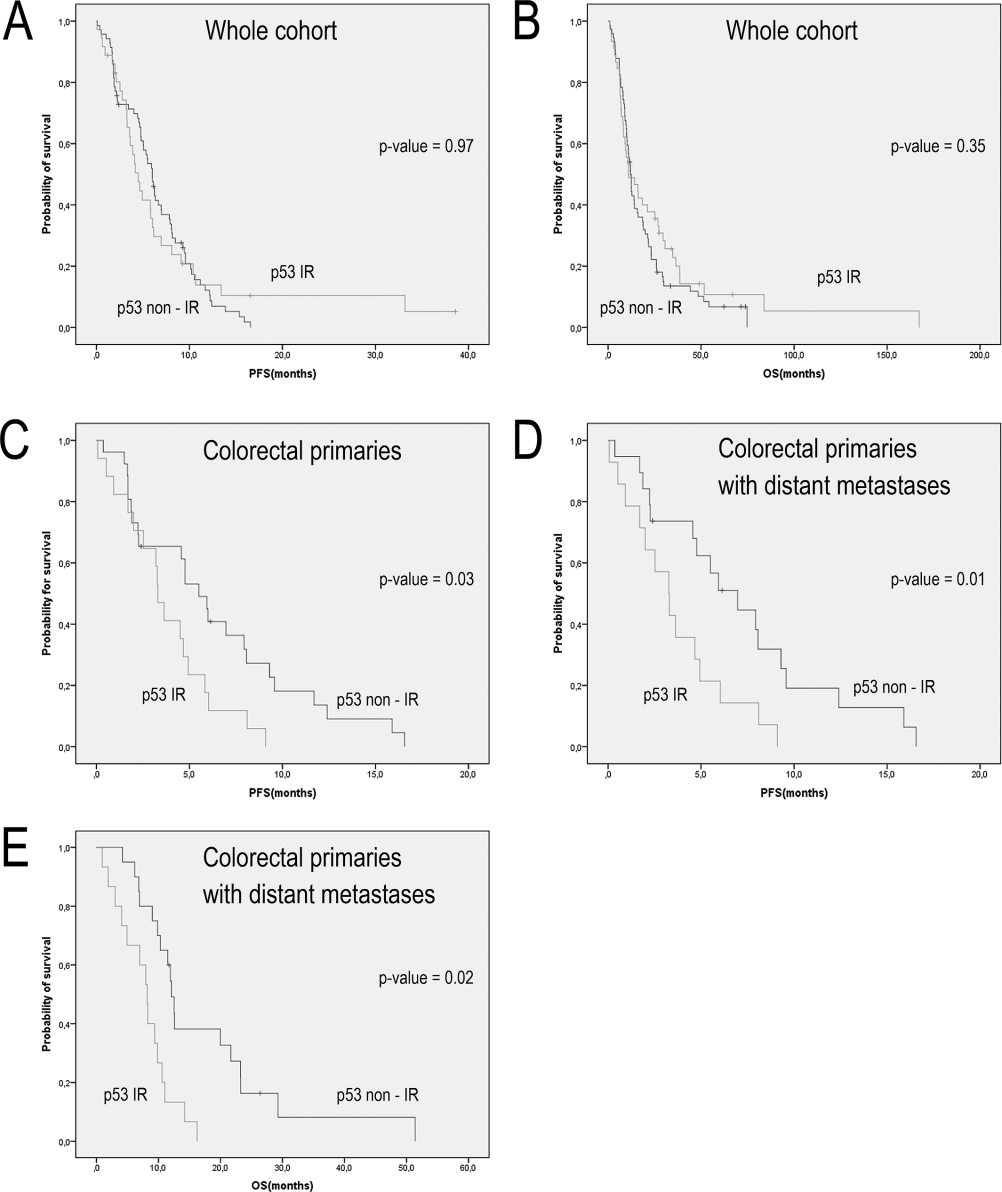
Kaplan-Meier curves. Kaplan-Meier survival curves for GEP-NECs divided according to primary tumor origin and p53 immunoreactivity. (A) Progression-free survival (PFS) for the complete cohort of 124 patients, p = 0.97. (B) Overall survival (OS) for the complete cohort of 124 patients, p = 0.54. (C) PFS for colorectal patients p = 0.03 (D) and (E) PFS and OS for colorectal patients with distant metastases, p = 0.01 and p = 0.02 respectively.

Kaplan-Meier analysis, dichotomized for p53 immunoreactivity, including all 124 patients did not show any differences in PFS and OS (Fig 3A and 3B, p = 0.97 and p = 0.54 respectively). When dividing the cohort into patients with or without distant metastases, no significant difference in survival could be detected with regard to expression of p53 (p = 0.97 for PFS and p = 0.61 for OS).

Patients with colorectal NECs expressing p53 protein had a shorter PFS compared to patients with non-IR tumors (3.3 vs. 5.1 months, p = 0.03) (Fig 3C). In the group of patients presenting colorectal tumors with distant metastases, both PFS and OS were shorter for patients with tumors IR for p53, 3.3 and 8.2 months respectively, compared to non-IR tumors with median PFS of 5.9 months and OS 12.0 months (Fig 3D and 3E, p = 0.01 and p = 0.02 respectively). Median PFS and OS for the tumor groups are shown in Table 3.

**Table 3:** Median PFS and OS in p53 immunoreactive and non-immunoreactive groups.

	p53 IR	p53 non-IR	p-value^◆^
**Whole cohort**			
Median PFS	4.1 months	6.0 months	0.97
Median OS	11.1 months	12.1 months	0.54
**Esophagus, stomach and pancreatic primaries**			
Median PFS	4.2 months	6.8 months	0.26
Median OS	26.4 months	15.8 months	0.13
**CUP**			
Median PFS	6.0 months	4.6 months	0.23
Median OS	26.9 months	9.6 months	0.16
**Colorectal primaries**			
Median PFS	3.3 months	5.1 months	0.03*
Median OS	8.7 months	12.0 months	0.09
**Colorectal primaries with distant metastases**			
Median PFS	3.3 months	5.9 months	0.01**
Median OS	8.2 months	12.0 months	0.02*

CUP, cancer with unknown primary; IR, immunoreactive; OS, overall survival; PFS, progression-free survival.

^**◆**^p-value obtained from Kaplan-Meier analysis.

*correlation is significant at the 0.05 level

**correlation is significant at the 0.01 level

In univariate analysis, p53 immunoreactivity showed a significant correlation with shorter PFS in colorectal patients (HR = 2.1, p = 0.03) and patients with colorectal tumors and distant metastases showing p53 immunoreactivity had a significantly shorter PFS and OS (HR = 2.6 and HR = 3.4, p = 0.03 and p = 0.02 respectively) compared to patients with non IR- tumors (Table 4).

**Table 4:** Univariate analysis of prognostic parameters.

	Progression-free Survival		Overall Survival	
	Hazard Ratio (95% CI)	p-value	Hazard Ratio (95% CI)	p-value
**Whole cohort**				
p53	0.9 (0.6–1.5)	0.97	1.1 (0.4–2.7)	0.89
Age	0.7 (0.5–1.0)	0.08	1.2 (0.5–3.1)	0.69
Ki67	1.2 (0.8–1.9)	0.40	1.8 (0.7–4.4)	0.19
LDH	2.2 (1.4–3.4)	<0.01**	1.6 (0.7–4.2)	0.27
Performance status	2.9 (1.8–4.8)	<0.01**	8.3 (2.5–27.4)	<0.01**
Specimen size	1.1 (0.8–1.8)	0.55	0.7 (0.4–1.2)	0.19
**Esophagus, stomach and pancreatic primaries**				
p53	0.6 (0.3–1.5)	0.28	0.6 (0.3–1.4)	0.26
Age	0.6 (0.3–1.2)	0.12	1.2 (0.6–2.4)	0.64
Ki67	1.3 (0.6–2.7)	0.57	1.5 (0.8–3.3)	0.23
LDH	1.9 (0.9–4.3)	0.09	2.7 (1.2–6.0)	0.02*
Performance status	3.9 (1.6–10.2)	<0.01**	7.4 (2.6–21.1)	<0.01**
Specimen size	0.9 (0.5–2.0)	0.97	0.7 (0.4–1.0)	0.03*
**CUP**				
p53	0.6 (0.2–1.5)	0.24	0.6 (0.2–1.3)	0.16
Age	0.9 (0.4–2.3)	0.98	1.2 (0.6–2.5)	0.64
Ki67	0.9 (0.4–1.8)	0.76	1.0 (0.5–2.0)	0.92
LDH	5.3 (0.7–40.2)	0.11	4.6 (1.5–14.1)	<0.01**
Performance status	2.9 (1.2–7.1)	0.02*	3.7 (1.5–9.1)	0.05*
Specimen size	1.4 (0.6–3.1)	0.47	0.6 (0.3–1.3)	0.20
**Colorectal primaries**				
p53	2.1 (1.1–4.1)	0.03*	1.7 (0.9–3.1)	0.09
Age	0.7 (0.4–1.3)	0.27	0.6 (0.3–1.2)	0.16
Ki67	0.9 (0.4–1.8)	0.76	1.0 (0.5–2.0)	0.92
LDH	1.6 (0.9–3.2)	0.14	2.9 (1.5–5.7)	<0.01**
Performance status	2.3 (1.0–5.8)	0.04*	3.2 (1.4–7.2)	<0.01**
Specimen size	1.4 (0.6–3.1)	0.47	0.6 (0.3–1.3)	0.20
**Colorectal primaries with distant metastases**				
p53	2.6 (1.2–5.7)	0.03*	3.4 (1.6–7.4)	0.02*
Age	0.6 (0.3–1.3)	0.21	0.5 (0.2–1.1)	0.07
Ki67	0.9 (0.4–2.0)	0.80	1.1 (0.5–2.3)	0.84
LDH	1.9 (0.9–4.2)	0.10	2.8 (1.3–6.2)	0.01**
Performance status	2.9 (1.1–7.8)	0.02*	3.2 (1.3–7.9)	0.01**
Specimen size	1.3 (0.5–3.4)	0.62	0.7 (0.3–1.7)	0.41

CUP, cancer with unknown primary; ECOG, the Eastern Cooperative Oncology Group consensus for performance status; LDH, lactate dehydrogenase. Hazard ratio (HR) and 95% confidence intervals (CI) obtained from Cox regression models.

*correlation is significant at the 0.05 level

**correlation is significant at the 0.01 level.

Dichotomized variables: p53 IR vs. non-IR, age ≤60 years vs. >60 years, Ki67 ≥55% vs. ≤55% LDH normal vs. high and performance status ECOG 0+1 vs. 2+3, specimen size needle biopsy vs. surgical specimen

In the group of colorectal patients with distant metastases, these associations remained significant in multivariate analysis adjusted for age, Ki67 index, LDH and the ECOG for performance status (Table 5). The range in PFS for foregut patients was 0.6–33.2 months, for colorectal patients 0.1–16.6 months and for CUP 0.1–38.6 months.

**Table 5:** Multivariate analysis of prognostic parameters.

	Progression-free Survival		Overall Survival	
	Hazard Ratio (95% CI)	P-value	Hazard Ratio (95% CI)	P-value
**Whole cohort**				
p53	1.5 (0.9–2.4)	0.12	1.0 (0.7–1.7)	0.73
Age	0.8 (0.5–1.2)	0.75	0.9 (0.6–1.3)	0.56
Ki67	0.9 (0.6–1.6)	0.97	0.9 (0.6–1.5)	0.80
LDH	2.0 (1.2–3.3)	<0.01**	2.4 (1.5–3.8)	<0.01**
Performance status	2.4 (1.4–4.2)	<0.01**	3.5 (1.9–6.1)	<0.01**
**Esophagus, stomach and pancreatic primaries**				
p53	1.2 (0.5–3.1)	0.70	1.1 (0.4–2.6)	0.89
Age	0.8 (0.3–1.8)	0.50	1.2 (0.5–3.1)	0.69
Ki67	1.5 (0.6–3.4)	0.38	1.8 (0.7–4.4)	0.19
LDH	1.6 (0.6–3.8)	0.33	1.6 (0.7–4.2)	0.27
Performance status	3.4 (1.1–9.8)	0.02*	8.3 (2.5–27.3)	<0.01**
**CUP**				
p53	1.8 (0.5–6.6)	0.41	1.2 (0.4–3.4)	0.76
Age	1.0 (0.4–23.0)	0.96	0.9 (0.4–2.3)	0.83
Ki67	0.6 (0.2–1.8)	0.35	0.3 (0.1–0.8)	0.02*
LDH	8.1 (0.8–80.9)	0.08	6.6 (1.8–24.2)	<0.01**
Performance status	1.9 (0.6–5.8)	0.24	3.6 (1.2–11.1)	0.02*
**Colorectal primaries**				
p53	2.0 (0.9–4.2)	0.08	1.4 (0.7–2.7)	0.32
Age	0.9 (0.4–1.8)	0.67	0.5 (0.3–1.1)	0.10
Ki67	0.7 (0.3–1.5)	0.41	0.8 (0.4–1.7)	0.57
LDH	1.6 (0.8–3.2)	0.17	2.8 (1.4–5.6)	<0.01**
Performance status	2.5 (0.9–6.7)	0.06	3.5 (1.3–9.5)	0.01**
**Colorectal primaries with distant metastases**				
p53	2.5 (1.1–5.8)	0.04*	3.0 (1.3–6.9)	<0.01**
Age	0.6 (0.3–1.5)	0.30	0.3 (0.1–0.9)	0.02*
Ki67	0.6 (0.3–1.5)	0.33	0.8 (0.3–1.8)	0.57
LDH	1.6 (0.7–3.8)	0.26	2.2 (0.9–5.0)	0.07
Performance status	4.1 (1.2–13.4)	0.02*	5.2 (1.6–16.9)	<0.01**

CUP, cancer with unknown primary; ECOG, The Eastern Cooperative Oncology Group consensus for performance status; LDH, lactate dehydrogenase. Hazard ratio (HR) and 95% confidence intervals (CI) obtained from Cox regression models.

*correlation is significant at the 0.05 level

**correlation is significant at the 0.01 level.

Dichotomized variables: p53 IR vs. non-IR, age ≤60 years vs. >60 years, Ki67 ≥55% vs. ≤55%, LDH normal vs. high and performance status ECOG 0+1 vs. 2+3, specimen size needle biopsy vs. surgical specimen.

### Association between p53 protein expression and response to treatment

DCR after platinum-based chemotherapy in this cohort was 67% (including complete response, partial response and stable disease).

In the group of patients with colorectal tumors with distant metastases, a positive correlation was found between p53 immunoreactivity and disease control by chemotherapy (Table 2). For patients with p53 IR tumors and distant metastases, disease control during chemotherapy was shorter than for those without p53 immunoreactivity. Only 44% of patients with p53 immunoreactivity showed disease control during chemotherapy treatment whereas 75% of the patients with tumors non-IR for p53 had disease control. This correlation was not seen in any of the other groups of primary tumors.

### Immunohistochemical controls

The p53 positive control showed IR tumor cells. When omitting the primary antibody, the immunoreactivity was completely abolished.

## Discussion

To our knowledge this is the largest study investigating p53 protein expression as a possible prognostic marker for GEP-NEC patients. We found that patients with colorectal NECs with distant metastases, expressing p53, had a significantly shorter PFS and OS, than those lacking p53 expression. This is in line with the correlation between performance status and response within the same group. Furthermore, the positive correlation between p53 expression and Ki67 indicates that p53 may be a marker for poorer prognosis [21].

In accordance with these findings, others have also suggested that p53 expression might be useful to distinguish between GEP-NECs and tumors belonging to the recently recognized category of well differentiated G3 NENs [3]. However, in this cohort, patients with esophageal, stomach, pancreatic and CUP tumors, showed no significant differences in PFS and OS when comparing patients with or without p53 immunoreactivity and median OS was longer than for the colorectal NEC patients, a finding which may be explained by a few long survivors in these small number groups of patients.

Two immunohistochemical staining patterns were observed for p53 expression. One subgroup of tumors exhibited scattered p53 IR tumors cells, where 20–40% of the tumor cells were IR, while another subgroup displayed densely packed tumor cells with 60–100% of tumor cells showing immunoreactivity. This is in accordance with a study of p53 in gastric cancer where similar staining patterns were observed. However, as in this study, no correlation was seen between these patterns and clinicopathological parameters [22], i.e. the clinical significance of these staining patterns remains unclear.

We found that tumor specimen size correlated positively with p53 protein expression in the way that surgical specimens were more frequently IR for p53 than needle biopsies. This finding is rather unexpected since patients undergoing surgery are more likely to have a better performance status and expected longer OS, and presence of p53 IR tumors is hypothesized to be associated with poorer outcome. One possible explanation might be that biopsies can be less representative than larger specimens.

Genetically, GEP-NECs show chromosomal instability. Alterations in the *TP53* gene have been observed in a significant number of cases, suggesting it may have a role in NEC development [21, 23]. Frequent mutations in the *TP53* gene was confirmed in a recent report, in which next-generation sequencing of 50 cancer-related genes in 23 NEC tumors showed several different mutations, but with the presence of *TP53* mutation in the majority of tumors. A high number of tumors with *TP53* mutation has also been demonstrated in pancreatic NEC-patients [3].

Results from a study of ovarian carcinoma demonstrate that p53 expression can be used as an alternate marker for *TP53* mutation in tumors, and consequently, provides a quicker screening method to choose appropriate treatment [17]. However, today there is no consensus on how scoring or pathological evaluation should be performed for p53 in neuroendocrine neoplasia [24].

There was a significant correlation between response to chemotherapy and p53 expression in patients with colorectal tumors with distant metastases; patients with a p53 expression had a poorer response to platinum-based chemotherapy. These patients also had a worse prognosis in general, and patients with p53 IR tumors scored lower on the ECOG scale for performance status.

In conclusion, p53 expression was correlated with poorer survival and poorer response to chemotherapy in patients with colorectal NECs, especially for those with distant metastases. To confirm the possible prognostic role of p53 in GEP-NECs, additional genetic studies are necessary. These should preferably be prospective and include genotyping to compare genetic information with p53 immunoreactivity and other possible biomarkers such as RB1, ATRX and DAXX to further investigate the relationship to clinical outcomes.
